# Burnout Among School Teachers During the COVID-19 Pandemic in Jazan Region, Saudi Arabia

**DOI:** 10.3389/fpsyg.2022.849328

**Published:** 2022-06-03

**Authors:** Ahmad Y. Alqassim, Mohammed O. Shami, Ahmed A. Ageeli, Mohssen H. Ageeli, Abrar A. Doweri, Zakaria I. Melaisi, Ahmed M. Wafi, Mohammed A. Muaddi, Maged El-Setouhy

**Affiliations:** ^1^Department of Family and Community Medicine, Faculty of Medicine, Jazan University, Jazan, Saudi Arabia; ^2^Faculty of Medicine, Jazan University, Jazan, Saudi Arabia; ^3^Jazan Health Affairs, Ministry of Health, Jazan, Saudi Arabia; ^4^Department of Physiology, Faculty of Medicine, Jazan University, Jazan, Saudi Arabia; ^5^Department of Community, Environmental and Occupational Medicine, Faculty of Medicine, Ain Shams University, Cairo, Egypt

**Keywords:** burnout, COVID-19, environmental health, Maslach Burnout Inventory, occupational health, Saudi Arabia, teachers

## Abstract

**Background:**

Burnout is a syndrome that results from stressors in the work environment that have not been successfully managed. The prevalence of burnout among schoolteachers was always controversial. COVID-19 pandemic added more stressors to teachers since they had to change their working styles in response to the pandemic lockdowns or curfews. In Saudi Arabia, the prevalence and determinants of burnout among school teachers were not measured by any other group during the COVID-19 pandemic stressors.

**Methods:**

A cross-sectional survey was conducted among 879 teachers in the Jazan region, Saudi Arabia, using the Maslach Burnout Inventory (MBI), during April 2021. Multistage cluster random sampling was used.

**Results:**

The mean age of the participants was 41.4 (±6.9) years. Male teachers represented 52.6% of the participants while females represented 47.4%. Most teachers showed burnout symptoms (69.6%). Consequences of burnout were observed, such as using psychotropic medications (4.6%), absenteeism (45.6%), lack of job satisfaction (7.7%), and changing schools (15.8%). Using the MBI scale, most teachers showed medium or high emotional exhaustion (57.6%), low depersonalization (62.2%), and low personal accomplishment (51.4%).

**Conclusion:**

Most teachers showed symptoms of burnout during the COVID-19 pandemic. Being an expert and ability to adapt to technology during the COVID-19 pandemic proved to effectively reduce burnout symptoms. Increasing incentives, early detection, and improving the work environment is recommended to diminish burnout consequences.

## Introduction

Burnout was defined as “a psychological syndrome emerging as a prolonged response to chronic interpersonal stressors on the job” (Maslach and Leiter, 2016). That is why it is considered as an occupational, psychological syndrome (Montero-Marin et al., 2009; Njim et al., 2019; Raymaker et al., 2020). It is usually developed in response to poor working circumstances, interpersonal interactions, poor work management, and administration (Moueleu Ngalagou et al., 2019; Ndongo et al., 2020; Civilotti et al., 2021; Goldstein and Alesbury, 2021; Losung et al., 2021; Sener et al., 2021; Wirkus et al., 2021). It usually affects medical professionals, teachers, police officers, army soldiers, as well as many other professionals (Burki, 2020; Ndongo et al., 2020; Goldstein and Alesbury, 2021; Grupe et al., 2021; Llorca-Pellicer et al., 2021; Mák et al., 2021; Márquez et al., 2021; Ribeiro et al., 2021; Righi et al., 2021; Wirkus et al., 2021). The syndrome is characterized by various symptoms ranging from emotional to psychiatric, cognitive, and psychosomatic symptoms with different severities (Bauer et al., 2006; Schilling et al., 2019; Lass-Hennemann et al., 2020; Civilotti et al., 2021; Juczynski and Oginska-Bulik, 2021; Oginska-Bulik and Juczynski, 2021; Righi et al., 2021). On the other hand, some studies detected relations between burnout and the personality types, emotional and spiritual intelligence (Pishghadam, 2012; Pishghadam et al., 2022). May be that why it could lead to premature retirement or even suicide in severe cases (Bauer et al., 2006; Christopher et al., 2020; Queiros et al., 2020; Harvey et al., 2021). Although some studies reported a low provenance of around 5% or less among teachers (Purdy et al., 1987; Maric et al., 2020; Pereira et al., 2021). Other studies reported a prevalence of 21% among Tunisian teachers and 24.5% among Iraqi teachers (Chennoufi et al., 2012; Al-Asadi et al., 2018).

Since December 2019, with the outbreak of the COVID-19 pandemic, burnout has increased dramatically not only among healthcare professionals but also among other professions (Afulani et al., 2021; Ma et al., 2021; Naldi et al., 2021; Oksanen et al., 2021). This would be due to changing work and lifestyles in response to the lockdowns and curfew that pushed the government to adopt working from home for some professions (Prado-Gasco et al., 2020; Price, 2020; Suliman et al., 2021).

The Kingdom of Saudi Arabia was also affected by the COVID-19 pandemic. Although the Ministry of Health implemented prevention strategies early in March 2020, the prevalence of COVID-19 increased to the extent that the Kingdom decided to go into lockdown for months and even partial or full curfew for sometimes (Alsofayan et al., 2020; MOH, 2020). In Saudi Arabia, the majority (73%) of teachers reported that they received online learning experiences and can support student learning through digital technology (Mann et al., 2020). However, challenges for teachers were raised during the COVID-19 pandemic, such as the inability to access modern technology, poor internet connection, and learner’s lack of motivation (Hakim, 2020). Consequently, schools and universities started employing online teaching strategies that were not previously used. These strategies put the teachers under different pressures to the extent that they might have experienced burnout (Raymaker et al., 2020). Moreover, data on burnout among school teacher in Saudi Arabia is scarce and nearly absent. That is why we hypothesized that these stressful conditions would positively affect the prevalence of burnout among school teachers. So, we aimed in this study to measure burnout prevalence and identify its determinants, among teachers in the Jazan region, in the far southwest of Saudi Arabia.

## Materials and Methods

### Study Design and Sample Size Calculation

We conducted a cross-sectional study involved 879 teachers of the Jazan region, in all levels of public and private schools in Saudi Arabia during April 2021.

The minimum sample size was calculated to be 844 teachers. It was calculated using the following formula (Pourhoseingholi et al., 2013):


$$n=\frac{{\text{Z}}^{2}\,P\,\,\left(1-P\right)}{d^{2}}$$

Where, *n* = Sample size.

*Z* = The level of confidence. We used level of confidence of 95% so we used *Z* = 1.96.

*P* = The expected prevalence. As the prevalence was unknown among teachers in Jazan we used 50%.

*d* = The precision (corresponding to the effect size). We used 5,

So $n=\frac{{\left(1.96\right)}^{2}\times \,50\times 50}{5^{2}}=384$

We duplicated the sample size to reduce the cluster effect and considered 10% as non-response rate. So the sample size was calculated to be 844.

### Sampling Technique and Study Population

**FIGURE 1:**
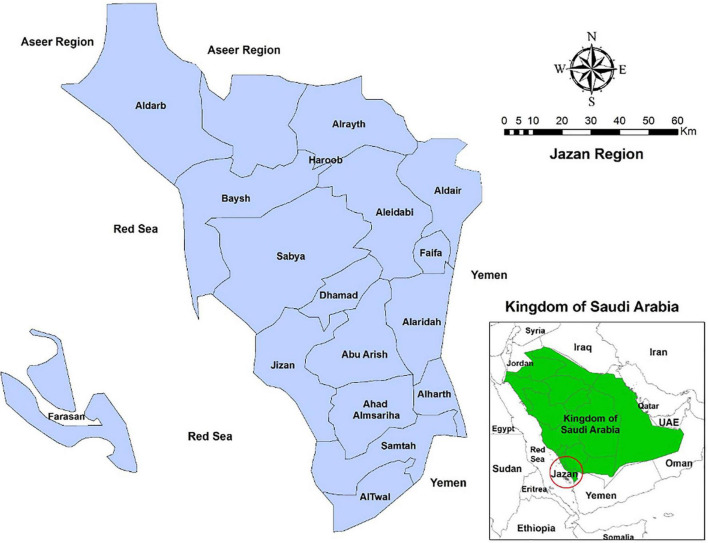
Jazan Province and its governorates.

We used a multistage cluster random sampling technique in this study. Jazan Province is one of the 13 provinces of Saudi Arabia. It is a highly populates (1.2 million) province that lies at the far southwest of the Kingdom. It is further subdivided into 13 governorates with Jazan City as a capital (Figure 1).

From educational point of view, Jazan province is divided into two educational directorates namely, Jazan and Sabya. Jazan directorate included six administrative offices and Sabya directorate included nine administrative offices. Each administrative office was supervising a number of schools of all levels (Primary, Intermediate, and High Schools).

We randomly chose three offices from each directorate using simple random sample technique. Subsequently, according to the school level, the schools in each education office were stratified into three strata (primary, intermediate, and secondary schools). Then using a systematic random technique, we chose two boys’ and two girls’ schools from each of the three school levels. So we ended up with 72 schools (24 primary, 24 middle, and 24 high schools). In the last stage, we invited all teachers (1,032) of all targeted schools to participate in the study. We received 879 completed questionnaires. The response rate was 85.2%.

### Study Tool

We used an online Google Forms Arabic questionnaire, including three main parts, to collect the data from the teachers.

The first part included questions regarding school teachers’ demographic and professional characteristics. These characteristics included; age, gender, marital status, number of children, level of education, residency, monthly income, school type, teaching level, his/her role/s in the school, number of working years, main daily teaching hours and main daily hours spent in preparation of lectures and other activities to be delivered to the students.

The second part of the questionnaire included questions on physical and mental health, social aspects, and future career plans. This section included questions about past history of treatment for depression or anxiety during the past 5 years, absenteeism during the COVID-19 pandemic, job satisfaction and planning to leave the school to another.

The third part included the validated Arabic version of Maslach Burnout Inventory (MBI) for educators (Abu-Hilal and Salameh, 1992; Maslach et al., 1996; Nesraoui and Zeroual, 2017; Chalghaf et al., 2019). This inventory is made up of 22 items on a 0–6 scale. These items were generated to measure the 3 components of burn out; emotional exhaustion, depersonalization, and personal accomplishment. We analyzed these components according to a previous study to describe each component as yes or no (Heidari and Gorjian, 2017). We then considered yes = 1 and no = 0. We then considered summation of the three components to get “No burnout” when the sum of the three components = 0, “Mild burnout” when the sum equal = 1, “Moderate burnout” hen the sum = 2 and “Severe burnout” when the sum = 3.

Before data collection, a pilot study was conducted to test whether the questionnaire’s wording was clear and understandable, as it was a translated version in Arabic. The reliability of the questionnaire was calculated using the Cronbach’s alpha test.

### Statistical Analysis

The collected data were coded, tabulated, and statistically analyzed using IBM SPSS statistics (Statistical Package for Social Sciences) software version 22.0, IBM Corp., Chicago, IL, United States, 2013 and Microsoft Office Excel 2007. Descriptive statistics were calculated for qualitative data as numbers and percentages. In contrast, inferential analyses were performed using the Chi-square test for differences between proportions and Fisher’s exact test for variables with small, expected numbers. A logistic regression model was used to identify independent factors affecting burnout and its subscales, and the selection of grades based on an in-depth analysis of each subscale. The level of significance was set at a *P*-value of < 0.050.

### Ethical Consideration

The study was approved by the Jazan University Scientific Research Ethics Committee (reference number REC42/1/014). Teachers who agreed to participate in the study read, understood, and accepted online consent forms. All participants were informed of their right to not participate or withdraw from the study at any time. The privacy and confidentiality of the data was maintained.

## Results

Performing Cronbach’s alpha test for our data showed that the emotional, personal accomplishment and for the whole scales were highly reliable (0.89, 0.87, and 0.90, respectively) and acceptable for the depersonalization scale (0.65). This data is not shown here but in the annex (1).

Table 1 shows the relationship between participants’ characteristics and their levels of burnout. A total of 879 teachers agreed to participate in the survey. Most of them (81.7%) were between 30 and 50 years. There were 462 male respondents, comprising 52.6% of the study sample, with a male-to-female ratio of 1.1. The majority (84.6%) of the study population were married, and 41.4% had more than three children. Approximately 88% of the participants had a bachelor’s degree, and 90.2% worked in governmental schools. Most teachers (78.2%, *n* = 687) worked only in teaching, while 157 (17.9%) had administrative roles. Regarding work experience, 376 (42.8%) had 10–20 years of teaching experience, 329 (37.4%) had more than 20 years of experience, and 174 (19.8%) had less than 10 years of experience. A total of 554 (63.0%) participants worked for 4–8 h a day. Participants aged 30–50 years, those who received salaries between SR 5,000 to 10,000, and those who lived in villages experienced significantly higher levels of burnout. Many demographic factors did not affect the levels of burnout among teachers. On the other hand, like age, residency, monthly income and the mean daily hours spent in preparation of the teaching materials were affecting the levels of the burnout among teachers. After using Bonferroni *post hoc* test for the chi square results, it was obvious that the middle age group (30–40 years showed higher levels of moderate and severe burnout. Moreover, low monthly income (<5,000 SR) was more associated with no to mild burnout while higher monthly income was more associated with higher levels of burnout. Finally, longer time spent in preparing the teaching materials was slightly associated with severe levels of burnout.

**TABLE 1:** The general characteristics of study participants according to burnout risk factors (*N* = 879).

Variables	Categories	All cases		Burnout			*P*-value
		(*N* = 879)	No (*N* = 268)	Mild (*N* = 440)	Moderate (*N* = 131)	Severe (*N* = 40)	
Age grades (years) (*n*, %)	<30	34 (3.9%)	12 (35.3%)^a^	18 (52.9%)^a^	3 (8.8%)^a^	1 (3.0%)^a^	0.016*^#^
	30–40	299 (34.0%)	88 (29.4%)^a^	131 (43.8%)^a^	66 (22.1%)^b^	14 (4.7%)^ab^	
	40–50	419 (47.7%)	128 (30.5%)^a^	222 (53.0%)^a^	49 (11.7%)^a^	20 (4.8%)^a^	
	50–60	127 (14.4%)	40 (31.5%)^a^	69 (54.3%)^a^	13 (10.2%)^a^	5 (4.0%)^a^	
Gender (*n*, %)	Male	462 (52.6%)	141 (30.5%)	225 (48.7%)	75 (16.3%)	21 (4.5%)	0.679^#^
	Female	417 (47.4%)	127 (30.5%)	215 (51.5%)	56 (13.4%)	19 (4.6%)	
Marital status	Single	102 (11.6%)	38 (37.3%)	48 (47.1%)	7 (6.8%)	9 (8.8)	0.054^§^
	Married	744 (84.6%)	220 (29.6%)	376 (50.5%)	118 (15.9%)	30 (4.0%)	
	Divorced/Widow	33 (3.8%)	10 (30.3%)	16 (48.5%)	6 (18.2%)	1 (3.0%)	
Number children	None	169 (19.2%)	60 (35.5%)	76 (45.0%)	21 (12.4%)	12 (7.1%)	0.158^#^
	1–3	346 (39.4%)	104 (30.1%)	170 (49.1%)	60 (17.3%)	12 (3.5%)	
	>3	364 (41.4%)	104 (28.6%)	194 (53.3%)	50 (13.7%)	16 (4.4%)	
Education	Postgraduate	37 (4.2%)	17 (45.9%)	14 (37.8%)	4 (10.8%)	2 (5.5%)	0.128^#^
	Bachelor	773 (87.9%)	236 (30.6%)	383 (49.5%)	117 (15.1%)	37 (4.8%)	
	Lower	69 (7.8%)	15 (21.8%)	43 (62.3%)	10 (14.5%)	1 (1.4%)	
Residence	City	405 (46.1%)	140 (34.6%)^a^	199 (49.1%)^a^	51 (12.6%)^a^	15 (3.7%)^a^	0.044*^#^
	Village	474 (53.9%)	128 (27.0%)^a^	241 (50.8%)^a^	80 (16.9%)^a^	25 (5.3%)^a^	
Monthly income in Saudi riyals (1 USD = 3.75 SR)	<5,000	70 (8.0%)	25 (35.7%)^a^	29 (41.4%)^a^	13 (18.6%)^a^	3 (4.3%)^a^	0.002*^#^
	5,000–10,000	100 (11.4%)	23 (23.0%)^ab^	66 (66.0%)^b^	8 (8.0%)^a^	3 (3.0%)^ab^	
	10,000–15,000	477 (54.3%)	134 (28.1%)^a^	236 (49.5%)^ab^	87 (18.2%)^b^	20 (4.2%)^ab^	
	≥15,000	232 (26.4%)	86 (37.1%)^a^	109 (47.0%)^ab^	23 (9.9%)^b^	14 (6.0%)^ab^	
School type	Governmental	793 (90.2%)	234 (29.5%)	403 (50.8%)	119 (6.4%)	37 (4.7%)	0.285^#^
	Private	86 (9.8%)	34 (39.5%)	37 (43.0%)	12 (14.0%)	3 (3.5%)	
Teaching level	Primary	341 (38.8%)	102 (29.9%)	180 (52.8%)	48 (14.1%)	11 (3.2%)	0.579^#^
	Preparatory	258 (29.4%)	76 (29.5%)	123 (47.6%)	44 (17.1%)	15 (5.8%)	
	High school	280 (31.9%)	90 (32.1%)	137 (48.9%)	39 (13.9%)	14 (5.1%)	
Role	Teaching	687 (78.2%)	213 (31.0%)	334 (48.6%)	108 (15.7%)	32 (4.7%)	0.353^#^
	Administration	157 (17.9%)	45 (28.7%)	88 (56.1%)	16 (10.2%)	8 (5.0%)	
	Both	35 (4.0%)	10 (28.6%)	18 (51.4%)	7 (20.0%)	0 (0.0%)	
Working years	<10.0	174 (19.8%)	60 (34.5%)	75 (43.1%)	33 (19.0%)	6 (3.4%)	0.137^#^
	10.0–<20.0	376 (42.8%)	105 (27.9%)	195 (51.8%)	60 (16.0%)	16 (4.3%)	
	≥20.0	329 (37.4%)	103 (31.3%)	170 (516%)	38 (11.6%)	18 (5.5%)	
Mean of daily teaching hours	<4.0	195 (22.2%)	65 (33.4%)	97 (49.7%)	24 (12.3%)	9 (4.6%)	0.870^#^
	4.0–<8.0	554 (63.0%)	168 (30.3%)	274 (49.5%)	87 (15.7%)	25 (4.5%)	
	≥8.0	130 (14.8%)	35 (26.9%)	69 (53.1%)	20 (15.4%)	6 (4.6%)	
Mean of daily preparation hours	<1.0	304 (34.6%)	91 (30.0%)^a^	170 (55.9%)^a^	35 (11.5%)^a^	8 (2.6%)^a^	0.023*^#^
	1.0–<2.0	285 (32.4%)	95 (33.3%)^a^	134 (47.0%)^a^	44 (15.4%)^a^	12 (4.2%)^a^	
	≥2.0	290 (33.0%)	82 (28.3%)^a^	136 (46.9%)^a^	52 (17.9%)^a^	20 (6.9%)^a^	

^#^Chi square test. §Fisher’s exact test. *Significant (<0.05). Homogenous groups had the same legend “a, b” based on post hoc Bonferroni test.

Table 2 shows the variables related to burnout among teachers, considering its severity. Among participating teachers (*n* = 879) in the Jazan region, 440 (50.1%) experienced mild burnout, 131 (14.9%) experienced moderate burnout, and 40 (4.6%) experienced severe burnout. No burnout was recorded in 268 (30.5%) teachers. All the factors used were significantly associated with burn out. However, after using *post hoc* Bonferroni test we find that those who were still using antipsychotic drugs, those who had more than twice absenteeism, those who not or never satisfied with their jobs and those who were looking to change their schools were experiencing more moderate to severe burnout.

**TABLE 2:** Consequences of burnout among teachers considering its severity.

Variables	Categories	All cases (*N* = 879)	Burnout				*P*-value
			No (*N* = 268)	Mild (*N* = 440)	Moderate (*N* = 131)	Severe (*N* = 40)	
Treated with antipsychotic (during the past 5 years)	Yes, still	10 (1.1%)	1 (10.0%)^a^	5 (50.0%)^ab^	2 (20.0%)^ab^	2 (20.0%)^b^	0.023*^§^
	Yes, stopped	30 (3.4%)	6 (20.0%)^a^	14 (46.7%)^a^	6 (20.0%)^a^	4 (13.3%)^a^	
	Never	839 (95.4%)	261 (31.1%)^a^	421 (50.2%)^a^	123 (14.6%)^ab^	34 (4.1%)^b^	
Absenteeism (during the COVID-19 pandemic)	None	478 (54.4%)	158 (33.1%)^a^	251 (52.5%)^a^	57 (11.9%)^b^	12 (2.5%)^b^	0.001*^#^
	Once	105 (11.9%)	27 (25.7%)^a^	54 (51.4%)^a^	19 (18.1%)^a^	5 (4.8%)^a^	
	Twice	103 (11.7%)	30 (29.2%)^a^	50 (48.5%)^a^	19 (18.4%)^a^	4 (3.9%)^a^	
	>Twice	193 (22.0%)	53 (27.5%)^a^	85 (44.0%)^a^	36 (18.7%)^ab^	19 (9.8%)^b^	
Job satisfaction	Very satisfied	490 (55.7%)	185 (37.7%)^a^	260 (53.1%)^b^	38 (7.8%)^c^	7 (1.4%)^c^	<0.001*^#^
	Satisfied	321 (36.5%)	82 (25.5%)^a^	152 (47.4%)^a^	69 (21.5%)^b^	18 (5.6%)^ab^	
	Not satisfied	46 (5.2%)	1 (2.2%)^a^	16 (34.8%)^b^	19 (41.3%)^c^	10 (21.7%)^c^	
	Never satisfied	22 (2.5%)	0 (0.0%)^a^	12 (54.6%)^b^	5 (22.7%)^c^	5 (22.7%)^c^	
Change school	Yes	139 (15.8%)	30 (21.6%)^a^	61 (43.9%)^a^	36 (25.9%)^b^	12 (8.6%)^b^	<0.001*^#^
	No	562 (63.9%)	186 (33.1%)^a^	298 (53.0%)^a^	63 (11.2%)^b^	15 (2.7%)^b^	
	May be	178 (20.3%)	52 (29.2%)^a^	81 (45.5%)^a^	32 (18.0%)^a^	13 (7.3%)^a^	

^#^Chi square test. ^§^ Fisher’s exact test. *Significant (<0.05). Homogenous groups had the same legend “a, b, c” based on post hoc Bonferroni test.

Table 3 shows the correlations between factors affecting burnout and its subscales. Regarding moderate/severe burnout, time spent usually in preparing lectures and activities to be delivered to the students in the next days (preparation time) of ≥2.0 h was a significant risk factor for moderate/severe burnout, while age >40 years and living in a village were significant protective factors. Regarding medium/high emotional exhaustion, a bachelor’s degree, teaching in preparatory schools (Teaching preparatory is grade 7–9), and time spent in preparing lectures and other activities to be delivered to the students (preparation time) >2 h were significant risk factors, while age >40.0 was a significant protective factor. The prevalence of emotional exhaustion was low in 373 (42.4%) participants, medium in 257 (29.2%) participants, and high in 249 (28.3%) participants. Depersonalization was low in 547 (62.2%) participants, medium in 174 (19.8%) participants, and high in 158 (18.0%) participants. Teaching for preparatory students, preparation time ≥2.0 h, and being male were significant risk factors, while residing in a village was a significant protective factor. Personal accomplishment was low in 452 (51.4%) participants, medium in 142 (16.2%) participants, and high in 285 (32.4%) participants. Notably, salary ≥15000.0 was a trigger factor for medium and high accomplishment.

**TABLE 3:** Logistic regression analysis for factors affecting burnout and its subscales.

Factors	β	SE	*P*	OR (95% CI)
**Moderate/severe burnout versus no and lower grades of burnout**				
Age >40 years	−0.57	0.17	0.001*	0.57 (0.40–0.80)
Residence in a village	−0.37	0.18	0.034*	0.69 (0.49–0.97)
Preparation ≥2.0	0.53	0.18	0.003*	1.70 (1.20–2.41)
Constant	−1.124	0.156	<0.001*	
**Medium/high emotional exhaustion versus no and lower grades of exhaustion**				
Bachelor education	0.50	0.21	0.020	1.64 (1.08–2.49)
Teaching preparatory	0.30	0.16	0.049	1.36 (1.00–1.84)
Preparation >2 h	0.30	0.15	0.009	1.48 (1.101–1.98)
Age groups >40.0	−0.35	0.15	0.015	0.70 (0.53–0.94)
Constant	−0.12	0.23	0.598	
**High depersonalization versus no and lower grades of depersonalization**				
Teaching preparatory	0.64	0.19	0.001*	1.89 (1.31–2.73)
Preparation ≥2.0	0.61	0.19	0.001*	1.85 (1.27–2.69)
Male	0.42	0.19	0.027*	1.52 (1.05–2.19)
Residence in a village	−0.69	0.19	<0.001*	0.50 (0.35–0.72)
Constant	−1.90	0.20	<0.001*	
**Medium/high accomplishment versus no and lower grades of accomplishment**				
Salary ≥15000.0	0.38	0.15	0.013*	1.47 (1.08–1.98)
Constant	−0.16	0.08	0.045*	

β, regression coefficient; SE, standard error; OR, odds ratio; CI, confidence interval. *Significant.

## Discussion

Many researchers used Maslach Burnout Inventory for educators all over the world. The inventory was reputedly validated in it original English language and in different languages including the Arabic language. We also validated the Arabic version of the inventory in our study. Our validation went with most of the other validation when the inventory showed higher Cronbach’s alpha for the subsections of the emotional and personal accomplishment, 0.89 and 0.87, respectively. The inventory as a whole also showed high Cronbach’s alpha (0.90). The only subscale that was questionable was the depersonalization subsection (Cronbach’s alpha 0.65). We presented the data of the Cronbach’s alpha tests in annex 1. Our results were in agreement of most of those who tested the validation of the inventory where always depersonalization subscales shoed the lowest Cronbach’s alpha (Abu-Hilal and Salameh, 1992; Shan and Gerstenberger, 2017; Szigeti et al., 2017; Chalghaf et al., 2019; Amri et al., 2021).

This study measured burnout and identified its determinants among teachers in the Jazan region in the far southwest of Saudi Arabia during the COVID-19 pandemic. Unfortunately, we could not find any published data on burnout among school teachers in Saudi Arabia, particularly in the Jazan region. However, few reports have been published in Tunisia Iraq and Iran (Chennoufi et al., 2012; Pishghadam, 2012; Pishghadam et al., 2014, 2022; Al-Asadi et al., 2018). That is why we included big sample of teachers with different experiences, school levels, residency, income, and both genders. Our study found that most teachers (69.6%) experienced different levels of burnout during the COVID-19 pandemic. However, nearly half of them experienced mild (50.1%), and to lesser extent, moderate (14.9%) levels. On the other hand, only 4.6% of our participants experienced severe symptoms of burnout.

Our study showed that burnout among Saudi teachers was associated with several demographic and work-related factors. This is consistent with other studies (Maslach, 2001; Borrelli et al., 2014; Saloviita and Pakarinen, 2020). Age was related to burnout levels and specially, emotional exhaustion. Teachers aged 50 years or older were less likely to report severe forms of burnout. Logistic regression analysis showed that age was an independent predictor of self-reported burnout. The same was reported in a recent publication (Al-Asadi et al., 2018; Silva et al., 2021). In our opinion, it is logical that older age groups usually have a better teaching experience that can help them shifting from face-to-face teaching to online teaching easily and without too much stress. We were thinking that working from home and using online technology in teaching would affect older age groups, but it did not.

The relation between gender and burnout is controversial. In some studies, burnout rates were higher among male teachers, while other studies found a higher prevalence of burnout among women (Unterbrink et al., 2007; Al-Asadi et al., 2018; Aziz et al., 2018; Greinacher et al., 2019; Ptacek et al., 2019). In our study, being a male teacher was independently associated with higher depersonalization. However, we did not detect any gender differences in reporting burnout. This result was also reported by different other studies where burnout affects male and female teachers equally (Maslach, 2001; Deguchi et al., 2018; Castaldelli-Maia et al., 2019; Borgonovi and Han, 2021). Different studies have offered several explanations, including the role of coping skills among teachers of each gender, work expectations, and social support. We can also attribute these to cultural differences and the role of males and females in carrying out the burdens of life according to different cultures, and how this would change with a pandemic. Actually, in Saudi Arabia during the pandemic males and females were trying to help each other more and were having more time to stay home together as well.

Monthly income influenced the level of burnout experienced by participants. Teachers who were well paid felt significantly high accomplishment than underpaid teachers. This is consistent with the findings of other authors who concluded that a lack of reward is strongly related to a feeling of inefficiency (Maslach, 2001; Gan et al., 2019; Selamu et al., 2019; Li et al., 2020). This is mainly a matter of satisfaction, rather than the amount of money.

Regarding the workload, many studies have shown that workload (expressed in our study as long hours spent for preparation of materials to be delivered to the students and teaching preparatory students, who are at the beginning of the teen age and need more time to understand and do better job in studying) was closely related to burnout in the form of emotional exhaustion and depersonalization among teachers, including Saudi teachers (Santana et al., 2012; Chalghaf et al., 2019; Amri et al., 2020; Salvagioni et al., 2020; Cobo-Vazquez et al., 2021; Kalynychenko et al., 2021; Li et al., 2021; Melanda et al., 2021; Ribeiro et al., 2021). Moreover, the notion of burnout as a response to job demands has been supported by both self-reports of experienced stress and more objective assessments of demands, such as the number of students and number of working hours (Maslach, 2001; Perry et al., 2014; Peterson et al., 2019; Hart et al., 2020; Ndongo et al., 2020; Shah et al., 2021).

Although living in cities is usually more stressful, our results showed that burnout was more likely to affect teachers living in rural areas than those in urban areas. This finding is consistent with that of a few studies conducted during the COVID-19 pandemic (Xin et al., 2020; Mueller et al., 2021; Peterson et al., 2021; Righi et al., 2021; Simaes et al., 2021). This might be because compared with the cities, internet in the villages is poor; thus, the pressure on teachers living in villages, to use online teaching is greater and may be the cause of higher burnout. Additionally, the loss of social contact during the curfew was the principal cause of burnout during this period. Moreover, usually those living in the village would have less spiritual intelligence as well (Pishghadam et al., 2022). However, these findings should be interpreted cautiously and replicated after the pandemic.

Although some factors lost their significance when combined in multivariate logistic regression, such as the use of psychotropic medications, overall satisfaction, and absenteeism, they are still risk factors of burnout among school teachers. They comprise a network of interacting, contributing factors that substantiate each other and negatively impact teachers’ quality of daily and professional life. This study suggests that serious efforts have been made to detect burnout through periodic psychological assessment, especially during the pandemic or outbreak times.

The current study is valuable as it is the first study in Saudi Arabia, particularly in the Jazan region, to use a validated scale (MBI) to measure burnout levels among teachers. Additionally, the results of this study are significant because it included a large sample of teachers (879), working at a number of different levels of schools (72), selected using multistage cluster sampling, with nearly 1:1 male to female ratio.

### Limitations

The present study had some limitations that should be considered when interpreting and generalizing its findings. The study’s cross-sectional design could not explain the temporal relationship between the study variables, which is consistent with findings of other studies (Maric et al., 2020). Moreover, since the current study was conducted during the COVID-19 pandemic, and we did not have burnout data before the pandemic, our results can only be generalized to the exact situation. Some other burnout predictors were not studied in this study like the personality traits, emotional and spiritual intelligence as well as the conceptions of assessments (Pishghadam, 2012; Pishghadam et al., 2022). However, as the population in Jazan area are homogenous and the evaluation of the teachers is following the same rules, these factors would have no effect on burnout in this area but should be considered in further researches.

### Future Research

Other burnout predictors should be studied including the personality traits, emotional and spiritual intelligence as well as the conceptions of assessments. Moreover, intervention studies should be conducted to find out the best methods to alleviate the burnout among teachers.

## Conclusion

Although burnout affected most of the teaches but its severity was mild to moderate. However, teachers living rural area, with less facilities, expressed more burnout during the pandemic. In Saudi Arabia, men and women are equally affected by burnout. It is recommended to increase teacher salaries and incentives, especially during stressful conditions, such as during a pandemic. It is good to repeat this work, especially after the pandemic, taking into consideration studying the personality traits, emotional and spiritual intelligence to have better information that would help in alleviating burnout symptoms among teachers.

## Data Availability Statement

The datasets generated and analyzed during the current study are available from the corresponding author upon reasonable request.

## Ethics Statement

The studies involving human participants were reviewed and approved by the Jazan University Scientific Research Ethics Committee. The patients/participants provided their written informed consent to participate in this study.

## Author Contributions

MS, AAA, MA, AD, ZM, AW, and MM contributed to the data and references collection, and helped in writing the first draft and tables. All authors contributed to the article and approved the submitted version.

## Conflict of Interest

The authors declare that the research was conducted in the absence of any commercial or financial relationships that could be construed as a potential conflict of interest.

## Publisher’s Note

All claims expressed in this article are solely those of the authors and do not necessarily represent those of their affiliated organizations, or those of the publisher, the editors and the reviewers. Any product that may be evaluated in this article, or claim that may be made by its manufacturer, is not guaranteed or endorsed by the publisher.
