# Supplementary material for: Burnout Among School Teachers During the COVID-19 Pandemic in Jazan Region, Saudi Arabia
Source: Front Psychol. 2022 Jun 3;13:849328. doi: 10.3389/fpsyg.2022.849328 (PMC9205293; doi:10.3389/fpsyg.2022.849328)
Supplement: Supplementary file 1 [file Data_Sheet_1.pdf]

```
Your temporary usage period for IBM SPSS Statistics will expire in 5076 days.

GET
  FILE='C:\Users\melsetouhy\Documents\INJURY\MOUSTFA ELHUSSAINI FILES\FES-I-Da
ta.sav'.
DATASET NAME DataSet1 WINDOW=FRONT.
GET
  FILE='C:\Users\melsetouhy\Desktop\Ahmad Elqassem\Teachers Burnout\Teachers D
ata-Jazan-Maged.sav'.
DATASET NAME DataSet2 WINDOW=FRONT.
DATASET ACTIVATE DataSet2.

SAVE OUTFILE='C:\Users\melsetouhy\Desktop\Ahmad Elqassem\Teachers Burnout\Teac
hers '+
    'Data-Jazan-Maged.sav'
  /COMPRESSED.
RELIABILITY
  /VARIABLES=MBI1 MBI2 MBI3 MBI6 MBI8 MBI13 MBI14 MBI16 MBI20
  /SCALE('ALL VARIABLES') ALL
  /MODEL=ALPHA
  /STATISTICS=DESCRIPTIVE SCALE CORR
  /SUMMARY=TOTAL.
```

Reliability

```
[DataSet2] C:\Users\melsetouhy\Desktop\Ahmad Elqassem\Teachers Burnout\Teacher
s Data-Jazan-Maged.sav
```

Scale: EMOTIONAL

Case Processing Summary

		N	%
Cases	Valid	879	100.0
	Excluded[a]	0	.0
	Total	879	100.0

a. Listwise deletion based on all variables in the procedure.

Reliability Statistics

Cronbach's Alpha	Cronbach's Alpha Based on Standardized Items	N of Items
.894	.893	9

Item Statistics

	Mean	Std. Deviation	N
MBI1	1.6780	1.96550	879
MBI2	3.4676	2.17011	879
MBI3	2.3732	2.13380	879
MBI6	2.3493	2.21863	879
MBI8	2.3675	2.18514	879
MBI13	1.0865	1.68723	879
MBI14	2.1490	2.15501	879
MBI16	1.6405	2.01543	879
MBI20	1.5495	2.05716	879

Inter-Item Correlation Matrix

	MBI1	MBI2	MBI3	MBI6	MBI8	MBI13	MBI14	MBI16
MBI1	1.000	.464	.393	.367	.457	.338	.417	.318
MBI2	.464	1.000	.631	.583	.615	.368	.549	.416
MBI3	.393	.631	1.000	.566	.598	.477	.533	.452
MBI6	.367	.583	.566	1.000	.608	.430	.568	.570
MBI8	.457	.615	.598	.608	1.000	.544	.672	.564
MBI13	.338	.368	.477	.430	.544	1.000	.584	.492
MBI14	.417	.549	.533	.568	.672	.584	1.000	.582
MBI16	.318	.416	.452	.570	.564	.492	.582	1.000
MBI20	.339	.344	.384	.386	.457	.448	.442	.401

Inter-Item Correlation Matrix

	MBI20
MBI1	.339
MBI2	.344
MBI3	.384
MBI6	.386
MBI8	.457
MBI13	.448
MBI14	.442
MBI16	.401
MBI20	1.000

Item-Total Statistics

	Scale Mean if Item Deleted	Scale Variance if Item Deleted	Corrected Item-Total Correlation	Squared Multiple Correlation	Cronbach's Alpha if Item Deleted
MBI1	16.9829	158.712	.513	.287	.893
MBI2	15.1934	147.496	.679	.546	.880
MBI3	16.2878	147.772	.688	.516	.879
MBI6	16.3117	145.730	.697	.523	.879
MBI8	16.2935	142.570	.779	.617	.872
MBI13	17.5745	158.844	.619	.441	.886
MBI14	16.5119	144.806	.743	.582	.875
MBI16	17.0205	152.098	.641	.461	.883
MBI20	17.1115	156.589	.528	.300	.892

Scale Statistics

Mean	Variance	Std. Deviation	N of Items
18.6610	188.001	13.71135	9

```
RELIABILITY
  /VARIABLES=MBI5 MBI10 MBI11 MBI15 MBI22
  /SCALE('ALL VARIABLES') ALL
  /MODEL=ALPHA
  /STATISTICS=DESCRIPTIVE SCALE CORR
  /SUMMARY=TOTAL.
```

Reliability

Scale: DEPERSONALIZATION

Case Processing Summary

		N	%
Cases	Valid	879	100.0
	Excluded[a]	0	.0
	Total	879	100.0

a. Listwise deletion based on all variables in the procedure.

Reliability Statistics

Cronbach's Alpha	Cronbach's Alpha Based on Standardized Items	N of Items
.652	.662	5

Item Statistics

	Mean	Std. Deviation	N
MBI5	.6268	1.41164	879
MBI10	.9408	1.65227	879
MBI11	1.0330	1.78645	879
MBI15	.7918	1.54467	879
MBI22	1.7884	2.10451	879

Inter-Item Correlation Matrix

	MBI5	MBI10	MBI11	MBI15	MBI22
MBI5	1.000	.340	.254	.235	.073
MBI10	.340	1.000	.704	.280	.233
MBI11	.254	.704	1.000	.281	.264
MBI15	.235	.280	.281	1.000	.153
MBI22	.073	.233	.264	.153	1.000

Item-Total Statistics

	Scale Mean if Item Deleted	Scale Variance if Item Deleted	Corrected Item-Total Correlation	Squared Multiple Correlation	Cronbach's Alpha if Item Deleted
MBI5	4.5540	24.471	.310	.137	.639
MBI10	4.2400	19.271	.606	.528	.503
MBI11	4.1479	18.627	.582	.512	.507
MBI15	4.3891	23.407	.335	.120	.630
MBI22	3.3925	21.332	.259	.080	.690

Scale Statistics

Mean	Variance	Std. Deviation	N of Items
5.1809	30.793	5.54914	5

```
RELIABILITY
  /VARIABLES=MBI4 MBI7 MBI9 MBI12 MBI17 MBI18 MBI19 MBI21
  /SCALE('ALL VARIABLES') ALL
  /MODEL=ALPHA
  /STATISTICS=DESCRIPTIVE SCALE CORR
  /SUMMARY=TOTAL.
```

Reliability

Scale: PERSONAL ACCOMPLISHMENT

Case Processing Summary

		N	%
Cases	Valid	879	100.0
	Excluded[a]	0	.0
	Total	879	100.0

a. Listwise deletion based on all variables in the procedure.

Reliability Statistics

Cronbach's Alpha	Cronbach's Alpha Based on Standardized Items	N of Items
.904	.905	8

Item Statistics

	Mean	Std. Deviation	N
MBI4	2.9044	2.47677	879
MBI7	3.5848	2.41376	879
MBI9	3.6940	2.33344	879
MBI12	3.6291	2.34475	879
MBI17	3.7201	2.41322	879
MBI18	4.1126	2.33178	879
MBI19	4.0239	2.28207	879
MBI21	3.6155	2.40208	879

Inter-Item Correlation Matrix

	MBI4	MBI7	MBI9	MBI12	MBI17	MBI18	MBI19	MBI21
MBI4	1.000	.471	.473	.336	.445	.389	.392	.390
MBI7	.471	1.000	.560	.470	.527	.484	.507	.511
MBI9	.473	.560	1.000	.605	.574	.555	.606	.476
MBI12	.336	.470	.605	1.000	.619	.624	.594	.535
MBI17	.445	.527	.574	.619	1.000	.688	.648	.619
MBI18	.389	.484	.555	.624	.688	1.000	.801	.665
MBI19	.392	.507	.606	.594	.648	.801	1.000	.682
MBI21	.390	.511	.476	.535	.619	.665	.682	1.000

Item-Total Statistics

	Scale Mean if Item Deleted	Scale Variance if Item Deleted	Corrected Item-Total Correlation	Squared Multiple Correlation	Cronbach's Alpha if Item Deleted
MBI4	26.3800	176.377	.515	.312	.909
MBI7	25.6997	170.201	.641	.437	.897
MBI9	25.5904	168.251	.705	.541	.891
MBI12	25.6553	168.812	.691	.524	.893
MBI17	25.5643	163.583	.761	.594	.886
MBI18	25.1718	164.418	.778	.710	.885
MBI19	25.2605	165.211	.784	.710	.885
MBI21	25.6689	166.591	.710	.555	.891

Scale Statistics

Mean	Variance	Std. Deviation	N of Items
29.2844	216.384	14.70999	8

```
RELIABILITY
  /VARIABLES=MBI1 MBI2 MBI3 MBI4 MBI5 MBI6 MBI7 MBI8 MBI9 MBI10 MBI11 MBI12 MB
I13 MBI14 MBI15 MBI16
    MBI17 MBI18 MBI19 MBI20 MBI21 MBI22
  /SCALE('ALL VARIABLES') ALL
  /MODEL=ALPHA
  /STATISTICS=DESCRIPTIVE SCALE CORR
  /SUMMARY=TOTAL.
```

Reliability

Scale: ALL VARIABLES

Case Processing Summary

		N	%
Cases	Valid	879	100.0
	Excluded[a]	0	.0
	Total	879	100.0

a. Listwise deletion based on all variables in the procedure.

Reliability Statistics

Cronbach's Alpha	Cronbach's Alpha Based on Standardized Items	N of Items
.899	.896	22

Item Statistics

	Mean	Std. Deviation	N
MBI1	1.6780	1.96550	879
MBI2	3.4676	2.17011	879
MBI3	2.3732	2.13380	879
MBI4	2.9044	2.47677	879
MBI5	.6268	1.41164	879
MBI6	2.3493	2.21863	879
MBI7	3.5848	2.41376	879
MBI8	2.3675	2.18514	879
MBI9	3.6940	2.33344	879
MBI10	.9408	1.65227	879
MBI11	1.0330	1.78645	879
MBI12	3.6291	2.34475	879
MBI13	1.0865	1.68723	879
MBI14	2.1490	2.15501	879
MBI15	.7918	1.54467	879
MBI16	1.6405	2.01543	879
MBI17	3.7201	2.41322	879
MBI18	4.1126	2.33178	879
MBI19	4.0239	2.28207	879
MBI20	1.5495	2.05716	879
MBI21	3.6155	2.40208	879
MBI22	1.7884	2.10451	879

Inter-Item Correlation Matrix

	MBI1	MBI2	MBI3	MBI4	MBI5	MBI6	MBI7	MBI8
MBI1	1.000	.464	.393	.264	.205	.367	.210	.457
MBI2	.464	1.000	.631	.445	.173	.583	.370	.615
MBI3	.393	.631	1.000	.366	.227	.566	.291	.598
MBI4	.264	.445	.366	1.000	.126	.352	.471	.331
MBI5	.205	.173	.227	.126	1.000	.319	.038	.267
MBI6	.367	.583	.566	.352	.319	1.000	.289	.608
MBI7	.210	.370	.291	.471	.038	.289	1.000	.357
MBI8	.457	.615	.598	.331	.267	.608	.357	1.000
MBI9	.185	.319	.223	.473	.061	.221	.560	.318
MBI10	.300	.314	.337	.135	.340	.359	.157	.439
MBI11	.292	.338	.392	.144	.254	.332	.122	.478
MBI12	.082	.210	.045	.336	-.028	.113	.470	.100
MBI13	.338	.368	.477	.151	.284	.430	.154	.544
MBI14	.417	.549	.533	.307	.195	.568	.341	.672
MBI15	.149	.123	.145	.048	.235	.179	.039	.181
MBI16	.318	.416	.452	.276	.259	.570	.273	.564
MBI17	.160	.341	.194	.445	.025	.211	.527	.233
MBI18	.081	.295	.161	.389	-.015	.142	.484	.164
MBI19	.124	.291	.182	.392	-.008	.149	.507	.239
MBI20	.339	.344	.384	.184	.221	.386	.140	.457
MBI21	.139	.264	.192	.390	-.016	.175	.511	.204
MBI22	.276	.261	.226	.229	.073	.290	.248	.350

Inter-Item Correlation Matrix

	MBI9	MBI10	MBI11	MBI12	MBI13	MBI14	MBI15	MBI16
MBI1	.185	.300	.292	.082	.338	.417	.149	.318
MBI2	.319	.314	.338	.210	.368	.549	.123	.416
MBI3	.223	.337	.392	.045	.477	.533	.145	.452
MBI4	.473	.135	.144	.336	.151	.307	.048	.276
MBI5	.061	.340	.254	-.028	.284	.195	.235	.259
MBI6	.221	.359	.332	.113	.430	.568	.179	.570
MBI7	.560	.157	.122	.470	.154	.341	.039	.273
MBI8	.318	.439	.478	.100	.544	.672	.181	.564
MBI9	1.000	.082	.095	.605	.114	.279	.050	.178
MBI10	.082	1.000	.704	-.027	.412	.348	.280	.391
MBI11	.095	.704	1.000	.005	.470	.389	.281	.417
MBI12	.605	-.027	.005	1.000	-.067	.132	.002	.048
MBI13	.114	.412	.470	-.067	1.000	.584	.232	.492
MBI14	.279	.348	.389	.132	.584	1.000	.140	.582
MBI15	.050	.280	.281	.002	.232	.140	1.000	.259
MBI16	.178	.391	.417	.048	.492	.582	.259	1.000
MBI17	.574	.003	.021	.619	.043	.267	.026	.149
MBI18	.555	-.008	.001	.624	-.022	.171	-.001	.085
MBI19	.606	.042	.029	.594	.015	.213	-.007	.117
MBI20	.137	.388	.412	.013	.448	.442	.165	.401
MBI21	.476	.050	.031	.535	.009	.201	-.013	.104
MBI22	.215	.233	.264	.184	.171	.337	.153	.314

Inter-Item Correlation Matrix

	MBI17	MBI18	MBI19	MBI20	MBI21	MBI22
MBI1	.160	.081	.124	.339	.139	.276
MBI2	.341	.295	.291	.344	.264	.261
MBI3	.194	.161	.182	.384	.192	.226
MBI4	.445	.389	.392	.184	.390	.229
MBI5	.025	-.015	-.008	.221	-.016	.073
MBI6	.211	.142	.149	.386	.175	.290
MBI7	.527	.484	.507	.140	.511	.248
MBI8	.233	.164	.239	.457	.204	.350
MBI9	.574	.555	.606	.137	.476	.215
MBI10	.003	-.008	.042	.388	.050	.233
MBI11	.021	.001	.029	.412	.031	.264
MBI12	.619	.624	.594	.013	.535	.184
MBI13	.043	-.022	.015	.448	.009	.171
MBI14	.267	.171	.213	.442	.201	.337
MBI15	.026	-.001	-.007	.165	-.013	.153
MBI16	.149	.085	.117	.401	.104	.314
MBI17	1.000	.688	.648	.085	.619	.187
MBI18	.688	1.000	.801	.147	.665	.192
MBI19	.648	.801	1.000	.184	.682	.239
MBI20	.085	.147	.184	1.000	.221	.370
MBI21	.619	.665	.682	.221	1.000	.285
MBI22	.187	.192	.239	.370	.285	1.000

Item-Total Statistics

	Scale Mean if Item Deleted	Scale Variance if Item Deleted	Corrected Item-Total Correlation	Squared Multiple Correlation	Cronbach's Alpha if Item Deleted
MBI1	51.4482	645.045	.455	.309	.896
MBI2	49.6587	618.494	.659	.586	.891
MBI3	50.7531	627.521	.583	.535	.893
MBI4	50.2218	620.478	.549	.391	.894
MBI5	52.4994	674.250	.247	.208	.900
MBI6	50.7770	623.351	.596	.550	.892
MBI7	49.5415	618.217	.586	.478	.893
MBI8	50.7588	615.659	.682	.648	.890
MBI9	49.4323	622.555	.570	.555	.893
MBI10	52.1854	656.987	.409	.553	.897
MBI11	52.0933	652.449	.424	.574	.897
MBI12	49.4972	638.385	.426	.555	.897
MBI13	52.0398	653.041	.446	.501	.896
MBI14	50.9772	620.881	.641	.603	.891
MBI15	52.3345	675.956	.199	.148	.901
MBI16	51.4858	635.214	.542	.492	.894
MBI17	49.4061	621.866	.554	.612	.894
MBI18	49.0137	628.444	.517	.720	.895
MBI19	49.1024	625.771	.555	.717	.893
MBI20	51.5768	640.871	.473	.403	.896
MBI21	49.5108	625.829	.522	.582	.894
MBI22	51.3379	644.345	.427	.266	.897

Scale Statistics

Mean	Variance	Std. Deviation	N of Items
53.1263	694.356	26.35064	22
